# Biochemical characterization of an *E. coli* cell division factor FtsE shows ATPase cycles similar to the NBDs of ABC-transporters

**DOI:** 10.1042/BSR20203034

**Published:** 2021-01-07

**Authors:** Sunanda Mallick, Ashish Kumar, Hiren Dodia, Cyrus Alexander, Dileep Vasudevan, Tushar Kant Beuria

**Affiliations:** 1 Infectious Disease Biology, Institute of Life Sciences, Nalco Square, Bhubaneswar, Odisha 751023, India; 2 Manipal Academy of Higher Education, Manipal, Karnataka 576104, India; 3 Regional Centre for Biotechnology, Faridabad, Haryana 121001, India

**Keywords:** ABC transporter, ATP hydrolysis, ATP-binding, FtsE dimer, FtsEX

## Abstract

The peptidoglycan (PG) layer is an intricate and dynamic component of the bacterial cell wall, which requires a constant balance between its synthesis and hydrolysis. FtsEX complex present on the inner membrane is shown to transduce signals to induce PG hydrolysis. FtsE has sequence similarity with the nucleotide-binding domains (NBDs) of ABC transporters. The NBDs in most of the ABC transporters couple ATP hydrolysis to transport molecules inside or outside the cell. Also, this reaction cycle is driven by the dimerization of NBDs. Though extensive studies have been carried out on the *Escherchia coli* FtsEX complex, it remains elusive regarding how FtsEX complex helps in signal transduction or transportation of molecules. Also, very little is known about the biochemical properties and ATPase activities of FtsE. Because of its strong interaction with the membrane-bound protein FtsX, FtsE stays insoluble upon overexpression in *E. coli*, and thus, most studies on *E. coli* FtsE (FtsE_Ec_) in the past have used refolded FtsE. Here in the present paper, for the first time, we report the soluble expression, purification, and biochemical characterization of FtsE from *E. coli*. The purified soluble FtsE exhibits high thermal stability, exhibits ATPase activity and has more than one ATP-binding site. We have also demonstrated a direct interaction between FtsE and the cytoplasmic loop of FtsX. Together, our findings suggest that during bacterial division, the ATPase cycle of FtsE and its interaction with the FtsX cytoplasmic loop may help to regulate the PG hydrolysis at the mid cell.

## Introduction

The bacterial cell division proceeds in two stages. In the first stage, chromosome segregation occurs, and in the second stage, the cell identifies the mid-cell position, where the division occurs. The constriction of cell envelope at the mid-cell assures the symmetric division of bacterial cells into two identical daughter cells. The reason for the occurrence of cytokinesis precisely at the mid-cell was unknown until the role of FtsZ in the assembly of the divisome was identified. FtsZ, a tubulin homolog, forms the core skeleton of the divisome by assembling into a ring-like structure at the mid-cell [1,2]. The two other essential cell division proteins, ZipA and FtsA help in stabilizing the Z-ring. FtsEX, an ABC transporter homolog [3], gets recruited to the Z-ring during the initial stages of the formation of the divisome complex [4,5]. In the FtsEX complex, FtsE forms the nucleotide-binding domain (NBD), and FtsX forms the transmembrane domain (TMD), and both are essential for the bacterial division. The ABC transporters primarily function as exporters or importers through remarkable conformational changes upon nucleotide-binding and hydrolysis. Apart from their role as a transporter, these family proteins help in the activation of other proteins [6]. For example, FtsEX activates amidases through EnvC, which performs peptidoglycan (PG) hydrolysis during bacterial cytokinesis [7]. Similar to other NBDs, FtsE possesses all the conserved regions, including the signature motifs, and the conserved residues involved in ATP-hydrolysis. The ATPase cycle is a necessary event for regulating the open and closed conformations of TMDs in ABC-transporters. Since TMD of the FtsEX complex is shorter, it may not show any open or closed conformations. Thus, if *E. coli* FtsE (FtsE_Ec_) shows any ATPase cycle, it may regulate the PG hydrolysis pathway at the mid-cell by regulating FtsX.

During cell division, there is a continuous requirement of PG hydrolysis at the mid-cell, and a regulated mechanism is essential to avoid breaches due to uncontrolled PG hydrolysis. Hence, the ATPase cycle by FtsE could be an essential event for the on/off mechanism of PG hydrolysis. Thus, it becomes critical to characterize the presence of the ATPase cycle and NBD-like functions of FtsE. In this work, we have used biochemical approaches and homology modeling to describe the functional and structural aspects of FtsE and its features similar to the NBDs of ABC-transporter. To date, no studies have reported FtsE_Ec_ protein in the soluble fraction, and most of the works published are done with refolded protein. Here, we were able to purify FtsE from the soluble fraction and validate its function precisely.

## Materials and methods

### Materials

HEPES, KCl, and sodium phosphate were from MP Biomedicals; LB broth and LB agar were from HiMedia, and MANT-ATP was from Invitrogen. All other chemicals used were of molecular biology grade and purchased from Sigma–Aldrich.

### Cloning and mutagenesis

The *ftsE* ORF was cloned at *EcoRI* and *SalI* restriction sites into a His-pBAD18 plasmid after amplifying from *E. coli* K12 genomic DNA. The clones were confirmed through colony PCR, restriction digestion, and finally, with dsDNA sequencing. Point mutations were generated by inverse PCR mutagenesis in *ftsE*. The plasmid with point mutations was treated with *DpnI* enzyme and incubated overnight at 37°C. The *DpnI-*treated PCR product was transformed into *E. coli* DH5α cells, and the mutations were confirmed by dsDNA sequencing.

### Expression and protein purification

The plasmid with wildtype and mutant *ftsE* were transformed into *E. coli* C41 competent cells. Initially, the expression was checked with different percentages of arabinose, starting from 0.01 to 0.1% in LB broth, which was followed by checking the protein in the soluble fraction. A good amount of protein was obtained in the soluble fraction with 0.01% arabinose. So, a maxi-culture was performed using the same arabinose concentration. Maxi-culture was performed by inoculating the overnight culture into 1 liter of fresh LB broth and incubated at 30°C in a shaker incubator till the OD_600_ reached 0.5. The protein expression was induced with 0.01% of arabinose at 16°C in shaker incubator for 16 h. The cells were harvested by centrifugation at 7000 rpm for 10 min at 4°C and resuspended with lysis buffer [25 mM sodium phosphate (pH 8.0), 300 mM KCl, 10 mM MgCl_2_, 0.01% NP-40 and 10% glycerol]. The suspended cells were homogenized with glass homogenizer for 1 h in ice and then passed through a pressure cell homogenizer four to five times. The cell lysate was then centrifuged at 40000×***g*** for 2 h. The clarified supernatant was incubated with pre-equilibrated Ni-NTA resin beads at 4°C for 1h. The resin beads were loaded into an empty gravity column, followed by washing of the beads with a wash buffer [25 mM HEPES (pH 7.5), 300 mM KCl and 25 mM imidazole]. Then the proteins were eluted with an elution buffer [25 mM HEPES (pH 7.5), 150 mM KCl and 250 mM imidazole). Fractions with relatively pure protein, as observed on an SDS/PAGE gel were pooled together and concentrated. Then the concentrated protein was subjected to size-exclusion chromatography (SEC) using a Superdex 75pg 16/600 column (GE Healthcare). The fractions from the second peak were collected and concentrated. Finally, the concentrated protein was once again passed through a Superdex 75pg 16/600 column, and the purified protein fractions were pooled together and concentrated. The concentrated protein was stored at −80°C. Similarly, the mutant FtsE proteins were also purified (Supplementary Figure S2A).

### Homology modeling of FtsE

The 3D structure of FtsE_Ec_ was made using a homology modeling technique. We used the Phyre2 template-based modeling [8] to model the structure of FtsE_Ec_. The modeling was done in intensive mode using FtsE_Ec_ protein sequence. Further, for atomic-level protein structure refinement, we used Fragment guided MD simulation [9] using an online interface of FG-MD (https://zhanglab.ccmb.med.umich.edu/FG-MD/). The model quality was further assessed by Ramachandran plot analysis using Phyre2 interface and RAMPAGE [10]. To further validate the correctness of the model, we used two other online homology modeling tools, including I-TASSER [11] and LOMETS [12,13]. All the structures were visualized and analyzed by PyMOL.

### ATPase assay

The ATPase assay was performed using Malachite Green Method as described earlier [14]. A 100-μl reaction mixture was set up with different concentrations (1, 5, and 10 μM) of the FtsE protein and incubated with 10 mM MgCl_2_ and 1 mM ATP at 37°C for 30 min. The reaction mixture was quenched with 70% perchloric acid (1:10 volume). Then, 950 μl of Malachite Green ammonium molybdate solution was added to 50 μl of the reaction mixture and incubated in the dark for 30 min at room temperature. Then the Pi released was calculated by measuring the absorbance at 650 nm and compared with the standard curve generated with sodium phosphate.

### Fluorescence binding assay

For determining the affinity of FtsE for ATP, 1 μM of MANT-ATP (a fluorescence analog of ATP) was incubated with varying concentrations of FtsE in a buffer containing 25 mM HEPES (pH 7.5), 50 mM KCl and 10 mM MgCl_2_. The reaction mixture was excited at 350 nm (the excitation wavelength for MANT-ATP), and the emission spectra were collected in the 380–550 nm wavelength range. Then the change in fluorescence intensity was used to calculate the dissociation constant using a double reciprocal plot [15].

### Job’s plot assay

The stoichiometry of FtsE and ATP was assessed using the continuous variation method. Then the change in mant-ATP fluorescence was observed by increasing the mant-ATP concentration and decreasing the FtsE concentration while keeping the total concentration constant at 1 μM. The reaction was carried out with 25 mM HEPES, pH 7.5, 50 mM KCl and 10 mM MgCl_2_.

### Analytical SEC

Analytical gel-filtration chromatography was carried out with a Superdex 200 increase 10/300 GL column (GE Healthcare). The column was pre-equilibrated with a buffer containing 20 mM HEPES (pH 8.0) and 50 mM KCl, and 100 μl of the protein sample was injected and allowed to pass through the column at 4°C with 0.5 ml/min flow rate in the same buffer. For molecular mass estimation of FtsE_Ec_, the standard proteins from the gel-filtration high molecular weight calibration kit (GE Healthcare) were passed through the column in the same buffer at 4°C, and molecular mass of the protein was calculated using *K*_av_ values.

### Circular dichroism spectroscopy

The near-UV CD (200–260 nm) spectrum was measured using a Circular Dichroism (CD) spectropolarimeter (JASCO-1500) to analyze the secondary structure content of FtsE_Ec_. The protein samples were diluted into 1× PBS to a concentration of 3 μM. The secondary structure content analysis was measured using CD multivariate SSE analysis option from SpectraManager software supplied with JASCO-1500 spectropolarimeter.

### Molecular docking of ATP

The docking was performed using AutoDock/Vina [16] with default parameters to get more accurate results. The pdbqt files for protein and ligand preparation and grid box were performed using a graphical user interface of AutoDock tools. AutoDock/Vina was further employed to dock ATP on to the protein, FtsE_Ec_. The program AutoDock/Vina considers both protein and ligand as rigid objects during docking and uses iterated local search global optimizer [16]. A total number of 10 poses were given, and the pose with the lowest binding affinity was extracted and aligned with FtsE to identify the residues involved in the interaction. The lowest binding affinity observed from docking was −8.7 kcal/mol from the first pose and was analyzed for further interpretation.

### Thermal stability assay

The thermal stability assay was performed by using FtsE protein purified from Ni-NTA resin. The protein was heated in a dry bath at different temperatures for 10 min. The heated protein samples were centrifuged at 40000×***g*** for 30 min. The supernatant and pellet were separated. To the pellet equal amount of buffer was added, and then both supernatant and pellet were loaded on SDS/PAGE separately and visualized with Coomassie stain.

### Yeast two-hybrid assay

Yeast two-hybrid (Y2H) was used to determine if FtsE interacts with itself following the previously established protocol [14]. The full-length FtsE was cloned in pGBT9 and pGAD424 separately. Both the plasmids were subsequently transferred into competent yeast cells and grown in histidine-deficient media.

### FtsE self-interaction and FtsE–FtsX interaction studies

For dimerization study, first of all the crude membrane fraction was obtained from *E. coli* (BL21) cell. The cells were lysed and centrifuged to remove the cytoplasmic proteins and then the pellet was used to isolate the membrane fraction using 0.1% Triton-X. A reaction mixture was set using purified His-FtsE and membrane fraction in the presence and absence of ATP and incubated in the ice for 1 h. Then the mixture was passed through cobalt resin and the eluted fraction was loaded in the SDS/PAGE without boiling, then followed by Western blot with anti-His monoclonal antibody. To check the interaction of FtsE with synthesized FtsX peptide (34 amino acids from cytoplasmic fraction), purified FtsE (10 μM) was incubated with FtsX peptide (20 μM) with 10 mM MgCl_2_ and in the presence or absence of 2 mM ATP. The reaction mixtures were incubated in ice for 30 min followed by glutaraldehyde cross-linking for another 30 min at room temperature. The samples were checked using an SDS/PAGE (18%).

## Results

### Purification of *E. coli* FtsE in native condition

FtsEX complex is a homolog of ABC-transporter in *E. coli*. The sequence alignment shows the presence of the conserved consensus sequence (Figure 2A) that confirms FtsE as the NBD in the FtsEX complex. Depending on the types of the TMD present, ABC transporters can function either as an exporter or an importer. However, FtsE neither functions as an exporter nor an importer. To understand how FtsE functions as an NBD, we evaluated its properties *in vitro*. Previously, FtsE protein from *M. tuberculosis* was expressed in *E. coli* and purified using denaturation and renaturation [17]. We followed the similar protocol to purify *E. coli*-FtsE (FtsE_Ec_). We obtained a good amount of purified protein using this method (Supplementary Figure S1A); however, the refolded FtsE was eluted near to the void volume when passed through an analytical SEC (Superdex 200 10/300 GL) (Supplementary Figure S1B). The CD analysis of the eluted fraction showed a poor secondary structure (data not shown) and very low ATPase activity suggesting non-functional protein (Supplementary Figure S3A). Therefore we modified our protocol to obtain functionally folded FtsE.

**Figure 1:**
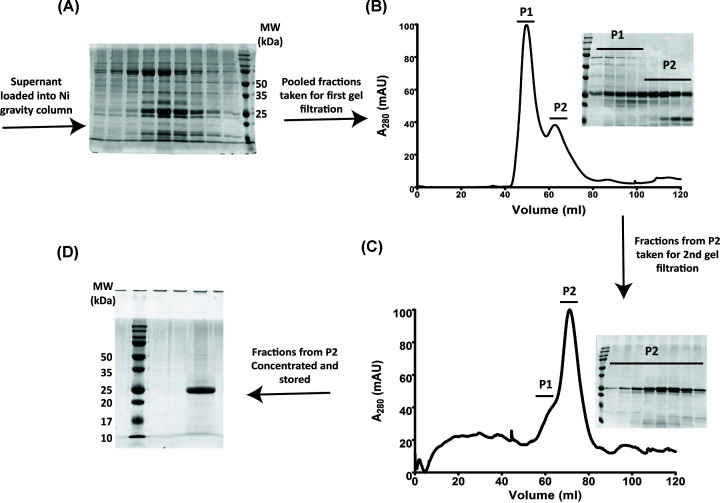
Purification of soluble FtsE: FtsE was purified using Ni-NTA affinity chromatography, followed by SEC (**A**) Shows the SDS/PAGE image for His-FtsE purified using Ni-NTA resin. (**B**) Shows the chromatogram and the SDS/PAGE image for purified His-FtsE using the Superdex 75pg column. (**C**) Shows the chromatogram and the SDS/PAGE image for the second round of purification of His-FtsE using the Superdex 75pg column. (**D**) Shows the purified soluble FtsE.

In the modified protocol, we used a His-pBAD18 vector system for the controlled expression of FtsE in *E. coli* C41 strains. The overexpression in this system was induced by 0.01% arabinose (w/v), and a large amount of FtsE was obtained in the soluble fraction. The purification was performed using Ni-NTA affinity chromatography, followed by two rounds of SEC (Figure 1A–C). To confirm the purified protein was FtsE, SDS/PAGE and Western blot was performed (Supplementary Figure S1C,D). In the analytical SEC, FtsE (Figure 4A) eluted as a monomer with an approximate molecular mass of 30.2 kDa (Figure 1D and Supplementary Figure S2B). The CD spectroscopy verified the presence of both α-helices and β-sheets (Supplementary Figure S2C), as predicted by secondary structure prediction tools. Our initial characterization showed that the purified FtsE_Ec_ is soluble; it has an intact secondary structure and remains as a monomer. This protein was further used for assessing its biochemical properties.

### Homology model of FtsE_Ec_ shows a typical NBD architecture

**Figure 2:**
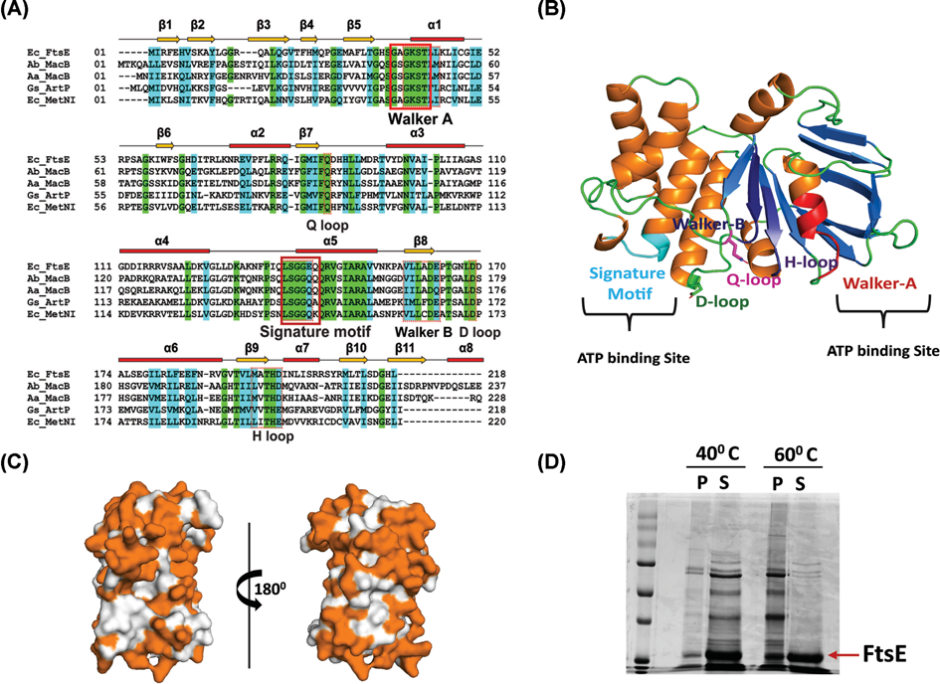
Sequence alignment of FtsE_EC_ with ABC-transporter family proteins and structure analysis using the predicted model of FtsE (**A**) Shows the sequence alignment of *E. coli* with the FtsE sequence from *Acinetobacter baumannii* MacB (Ab_MacB), *Aggregatibacter actinomycetemcomitans* MacB (Aa_MacB), *Geobacillus stearothermophilus* ArtP (Gs_ArtP), and *E. coli methionine* importer (Ec_MetNI). (**B**) The structure of FtsE_Ec_, showing all the conserved motifs within the structure (Red: Walker-A, Blue: Walker-B, Pink: Q-loop, Green: D-loop, Purple: H-loop, and Cyan: the signature motif). (**C**) The distribution of hydrophobic residues across the FtsE surface (regions colored in White, represents the hydrophobic residues). (**D**) SDS/PAGE image showing the thermal stability of the recombinant FtsE: P, pellet; S, supernatant.

To study the properties and functions of FtsE in *E. coli*, we evaluated the FtsE structure computationally. The multiple sequence alignment of FtsE_Ec_ showed ∼42% sequence identity with methionine importer, MetNI, from *E. coli* (strain K12). The other structural homologs of FtsE_Ec_ with more than 35% sequence identity include an ABC-transporter MacB from *Acinetobacter baumannii* (39.3%), a non-canonical ABC-transporter from *S. pneumoniae* (R6 strain) (36.4%), an ABC-transporter from *Aquifex aeolicus* (36.3%) and ArtP from *Geobacillus stearothermophilus*. The sequence alignment reveals that the consensus sequences of ABC-transporter family proteins such as Walker A, Walker B motif, Q-loop, D-loop, H-loop, and the signature motif (LSGGQ) are conserved in FtsE_Ec_ (Figure 2A) suggesting FtsE_Ec_ as a member of the ABC-transporter family. The structure of FtsE_Ec_ was predicted by comparative modeling using Phyre2 prediction server, which showed more than 90% confidence for the secondary structural elements [8]. Further validation of the predicted structure was performed using separate online modeling tools based on different algorithms such as I-TASSER [11] and LOMETS [12,13]. All the predicted structures showed secondary structures similar to that obtained by Phyre2, and therefore it was used for further analysis.

The structural analysis showed that FtsE_Ec_ contains 11 β-sheets and 8 α-helices. The β-strands are present as anti-parallel β-sheets arranged in two halves forming a pocket-like structure with α1 helix inserted inside it. The other α-helices lie on the other half of the structure. The Walker-A motif lies in α1 helix and loop connecting β5 and α1. Whereas, the Walker-B motif lies in the β8 strand (Figure 2A,B). We also observed more number of hydrophobic residues distributed over the surface of FtsE (Figure 2C). A higher distribution of hydrophobic residues over the surface may account for the overall higher stability of FtsE_Ec_ protein. Thus, the thermal stability of FtsE_Ec_ was checked by heating the protein sample at different temperatures. The data show that FtsE possesses higher thermal stability (Figure 2D), which was also verified using CD-spectroscopy (data not shown). Furthermore, the surface electrostatics distribution of the FtsE_Ec_ model showed an even charge distribution on both the faces (Supplementary Figure S2D).

### FtsE_Ec_ interacts with ATP and shows ATPase activity similar to NBDs

The ABC-transporters shows a conserved architecture, where two TMDs associate with each other to form translocon channel and substrate binding sites. The crystal structure of NBDs in the nucleotide-binding state or the whole transporter shows a symmetrical sandwich dimer, where two nucleotides are bound at the monomer–monomer interface of NBDs [18]. Two alternative models have been proposed for NBDs function; processive clamp or switch model and constant contact model [19]. In the processive clamp model, NBDs undergo a cycle of ATP binding followed by dimer formation, which further follows ATP hydrolysis and NBD dimer dissociation. Alternatively, in constant contact model, the NBDs always remain in the asymmetric state, and only one nucleotide stay tightly bound at a time [20].

**Figure 3:**
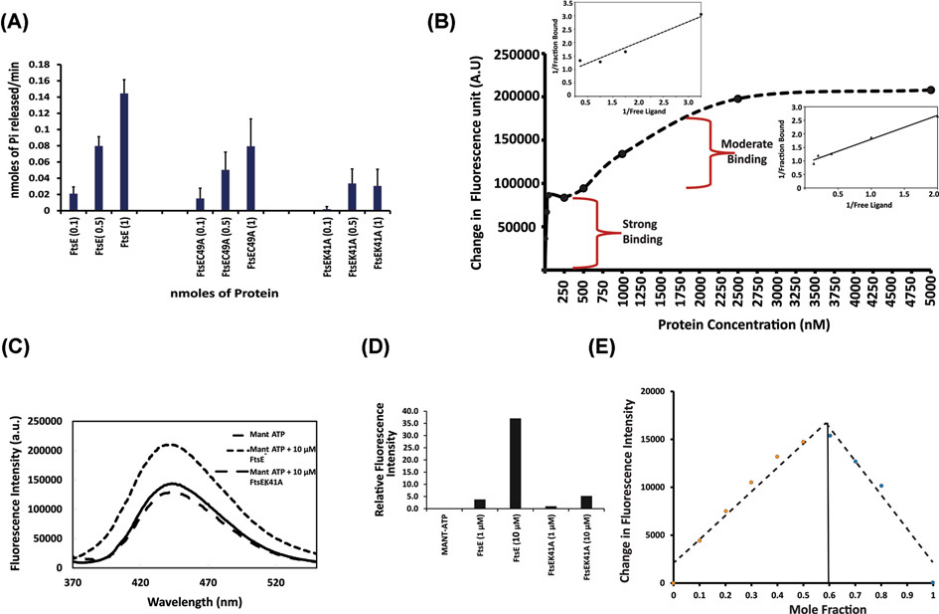
ATPase activity and ATP binding The ATPase activity and ATP-binding studies were performed as described in the method section. (**A**) Shows the ATPase activity of wildtype FtsE and the two mutant versions of FtsE (EC49A and EK41A). (**B**) ATP-binding affinity of FtsE, was performed using fluorescence binding assay. (**C**) Graph showing the fluorescence intensity of MANT-ATP in the presence of wildtype FtsE and ATP binding mutant of FtsE (K41A). (**D**) Dendrogram is showing the relative fluorescence intensity of wildtype and mutant FtsE. (**E**) Job’s plot showing the binding ratio of FtsE and ATP (Mant-ATP used for fluroscence excitation).

To identify the ATP-binding pattern of FtsE_Ec_ and its model of function, we performed fluorescence binding assay. FtsE_Ec_ was titrated against MANT-ATP, and the change in fluorescence intensity was used to calculate the dissociation constant. Our results indicated the presence of two binding sites in wildtype FtsE, with a high-affinity site (*K*_d_ = 21.5 ± 2.2 nM) and a moderate affinity site (*K*_d_ = 1.14 ± 0.24 μM) (Figure 3B). The results clearly showed that, like NBDs, the binding of ATP to FtsE_Ec_ is sequential. To verify the same, we determined the stoichiometry of the FtsE–MANT–ATP complex using the continuous variation method. Our result showed that more than one (number) ATP (∼1.5) interacted with FtsE (Figure 3E).

We also assessed the ATPase activity of wildtype, ATP binding-deficient mutants (K41A, C49A) and refolded FtsE *in vitro*. Wildtype FtsE_Ec_ showed an increase in ATPase activity in a concentration-dependent manner (Figure 3A). The rate of ATPase activity was found to be 0.14 ± 0.01 nmoles of Pi/nmoles of protein/min for wildtype FtsE. Whereas, the refolded FtsE_Ec_ showed much lower ATPase activity compared with the native FtsE_Ec_ (Supplementary Figure S3A). When the lysine residue that forms the Walker-A loop (K41 in *E. coli* FtsE) and binds to ATP was mutated to alanine (FtsE^K41A^), the ATPase activity was reduced by 50–90% (Figure 3A). The reduced ATPase activity suggests that mutation at a single site did not abolish the ATPase activity of FtsE_Ec_ completely. We also checked the ATP binding affinity for FtsE^K41A^, and it was found to be much lower than wildtype FtsE (Figure 3C,D). Similarly, mutation of cysteine (C49) to alanine (FtsE^C49A^), a residue reported to be involved in FtsE dimerization [21], showed a decreased ATPase activity (by 30%) compared with the wildtype FtsE_Ec_ (Figure 3A). Since cysteine residue is present adjacent to the Walker-A motif, a mutation of cysteine residue may lead to a decrease in the ATP binding and, eventually, ATPase activity.

### FtsE self-interaction and its interaction with FtsX

**Figure 4:**
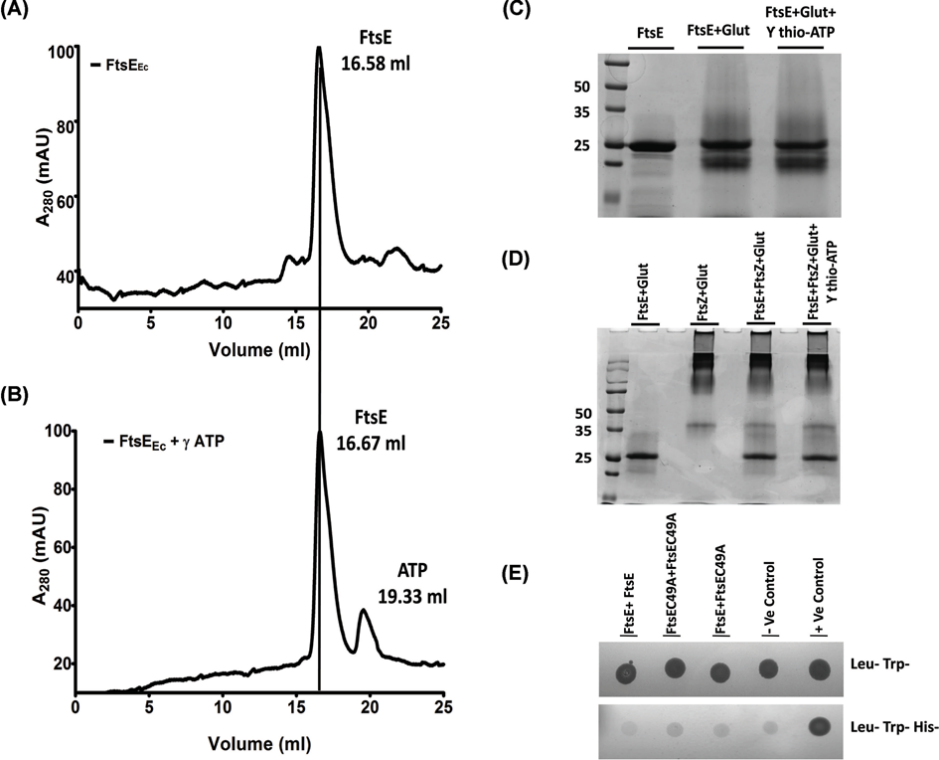
FtsE self-interaction FtsE self-interaction was performed using SEC and glutaraldehyde cross-linking. Analytical size-exclusion chromatogram for FtsE in the absence (**A**) and the presence of ATP (**B**) are shown. (**C**) The SDS/PAGE image, is showing glutaraldehyde cross-linking of FtsE in the presence and absence of γ-ATP. (**D**) The SDS/PAGE showing glutaraldehyde cross-linking of FtsE in the presence of FtsZ and γ-ATP. (**E**) Y2H experiment showing the FtsE self-interaction.

From ATP-binding and the ATPase assays with wildtype and mutant FtsE, we observed that more than one ATP-binding sites are present in FtsE_Ec_. To further probe whether ATP binds to the same residues as in other NBDs, we docked ATP over the FtsE_Ec_ monomer model using Autodock Vina [16]. The docking prediction shows that K41 of the Walker-A motif of FtsE_Ec_ monomer is involved in binding with ATP (Supplementary Figure S3C,D) and the corresponding lysine-residue in the Walker-A motif is a conserved residue for ATP-binding in NBDs. Thus, if FtsE_Ec_ forms a dimer, two ATP molecules could be present at the dimer interface, where each of the monomers would contribute one nucleotide. To check the possibility of FtsE_Ec_ forming a dimer experimentally, we first performed analytical SEC of FtsE_Ec_ with ATP and FtsE_Ec_ without ATP. SEC result did not show any difference in the elution profile or peak position in both the cases (Figure 4A,B). We also performed glutaraldehyde cross-linking and checked the samples on SDS/PAGE. Here again, FtsE_Ec_, both in the absence and presence of ATP, ran as a monomer and did not show any dimer formation (Figure 4C). We further performed a Y2H assay, which also showed no direct interaction between FtsE monomers (Figure 4E). Thus our results suggested that FtsE_Ec_ could not dimerize alone or in the presence of ATP. Next, we aimed to check whether any interacting partners are required for the dimerization of FtsE. FtsZ is known to interact with FtsE_Ec_ directly, and thus we performed a glutaraldehyde cross-linking experiment on FtsE_Ec_ in the presence of FtsZ and ATP. However, we did not see any FtsE dimers (Figure 4D). From the above results, we summarize that FtsE_Ec_ does not form a functional homodimer alone or in the presence of either ATP, FtsZ, or both.

**Figure 5:**
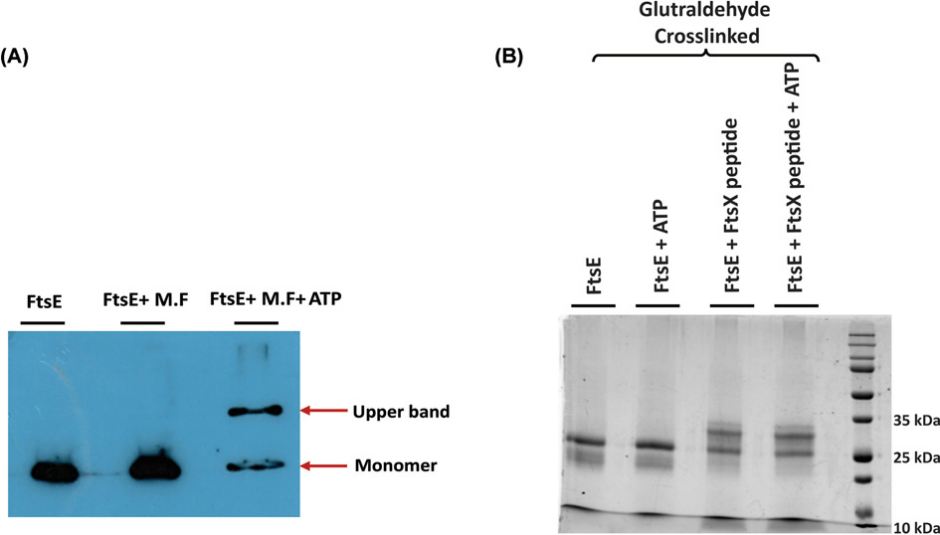
Interaction of FtsE with a cytoplasmic peptide of FtsX (**A**) Western blot with anti-His antibody, indicating FtsE-FtsX complex formation in the presence of *E. coli* membrane fraction. (**B**) An 18% SDS/PAGE showing glutaraldehyde cross-linking of FtsE and cytoplasmic loop of FtsX (FtsX-peptide).

Nevertheless, we could not ignore the possibility of the requirement of its interacting partner, FtsX, for forming a stable dimer. To check this possibility, we incubated His-purified FtsE in the presence of ATP and with the cytoplasmic fraction, or the membrane fraction of *E. coli* lysate. The sample was subjected to semi-denaturing SDS/PAGE and Western blotting using an anti-His antibody. Interestingly, FtsE showed two bands in SDS/PAGE, one at ∼26 kDa and second at ∼55 kDa, whereas, in the presence of the cytoplasmic fraction, FtsE ran as a monomer (Supplementary Figure S3B). Although the Western blot confirmed the presence of FtsE at both the positions, it did not conclude if the upper band is due to FtsE homodimer or due to the formation of a heterocomplex with any of its membrane partner such as FtsX (Figure 5A). To further confirm the involvement of FtsX, we synthesized a 34-amino acid peptide from the cytoplasmic loop of FtsX (TMD2 to TMD3), and the FtsE dimerization experiment was performed in its presence. Then after glutaraldehyde cross-linking, the samples were run on SDS/PAGE.

Interestingly, we did not observe any FtsE dimer; instead, we found that FtsE and FtsX peptide were interacting with each other and showing a shift in FtsE band to ∼30 kDa size (Figure 5B). Our results indicated that FtsE_Ec_ might not homodimerize in *in-vitro* condition, whereas, it may form a heterocomplex with FtsX even in the absence of ATP. Our results also suggested that FtsE–FtsX interaction is not dependent on ATP. However, the cases may be different during *in vivo*, and further investigation is required to understand if and how FtsE is forming the dimers.

## Discussion

FtsEX complex is an ABC transporter homolog, where FtsE is the NBD, and FtsX is the TMD. Unlike other ABC transporters, FtsEX is not involved in membrane transportation. The predicted structure of FtsX contains a large extra periplasmic loop suggesting its role in the signaling pathway [22]. During bacterial cell division, this extra periplasmic loop of FtsX interacts with EnvC that activates the amidases A and B and initiates PG hydrolysis at the mid-cell [7]. On the other hand, the ATPase activity of FtsE and its interaction with the cytoplasmic loop of FtsX regulates the PG hydrolysis. FtsEX complex does not transport anything. However, a significant question is whether FtsE functions like an NBD, i.e. whether it forms an active dimer and possesses ATPase activity.

To answer this question, we elucidated the NBD-like functions of FtsE_Ec_ *in vitro*. We initially purified FtsE_Ec_ from inclusion bodies by denaturation and refolding. However, analytical-SEC and CD spectroscopy showed that the refolded FtsE_Ec_ forms larger aggregates with minimal secondary structures. Thus, FtsE was further purified from the soluble fraction; this protein showed proper secondary structures and did not show any aggregation (Figure 4A, Supplementary Figure S2C). Presence of the hydrophobic residues on the surface can stabilize globular proteins and can increase the thermal stability of a protein [23]. In FtsE, more number of hydrophobic residues appear to be clustered along with polar residues on surface (Supplementary Figure S2D) that may increase its thermal stability. This was also evident from our result that showed higher thermal stability for purified FtsE (Figure 2D). The ATPase activity of the purified natively folded FtsE was much higher than that of the refolded FtsE (Supplementary Figure S3A). The homology model of FtsE_Ec_ showed an architecture similar to NBDs and suggested that it may form an active dimer (Figure 2B). However, the glutaraldehyde cross-linking, analytical-SEC experiments, and the Y2H assay showed that FtsE does not form any dimer *in vitro* either in the absence or presence of ATP. Our findings are similar to the results obtained from previous studies where FtsE from *S. pneumoniae* also did not form an active dimer with glutaraldehyde cross-linking [24]. Thus apart from ATP, FtsE might need its cognate partners FtsX or some other substrate for its dimerization. To verify this, we performed the dimerization of FtsE in the presence of *E. coli* membrane fraction and ATP.

Interestingly, in the presence of the *E. coli* membrane fraction, FtsE showed an upper band at the molecular size similar to FtsE homodimer or FtsE–FtsX hetero-complex (Figure 5A). To verify this, we designed a small peptide from the cytoplasmic loop of FtsX and performed the FtsE dimerization experiment. Our result showed that in the *in vitro* experiment condition, FtsE did not form any dimer, but it interacted with the FtsX-peptide (Figure 5B). This observation suggested that the shift in FtsE band to dimer position in the presence of the *E. coli* membrane fraction (Figure 5A) might be due to the formation of a hetero-complex of FtsE and FtsX. Our result confirmed that the cytoplasmic loop of FtsX is responsible for FtsE–FtsX interaction, which was initially a prediction.

We also observed that the FtsE–FtsX peptide interaction is not ATP-dependent. Although we were unable to show FtsE dimerization *in vitro*, it could be possible that our experiment did not mimic the exact required condition for FtsE dimerization. The experiment might require other components like an intact membrane or full-length FtsX. The dimerization of ABC transporters requires two ATP binding sites in each monomer, where the ATPs are shared among the FtsE_Ec_ monomers to facilitate the dimerization. Using fluorescence binding assay and Job’s plot assay, we observed that FtsE_Ec_ contains more than one ATP binding sites (Figure 3B–E). Lys^41^ is known to interact with ATP. Our molecular docking results also showed that Lys^41^ interacts with the three phosphates, similar to shown for other NBDs [25]. However, this FtsE mutant (K41A) still showed some ATPase activity, that further supports the presence of two ATP-binding sites in FtsE. In wildtype FtsE, we observed the presence of two ATP binding sites, one with higher affinity (25 nM) and another with a moderate affinity (1 μM). As Lys^41^ is the primary residue, we observed from our docking results, and from literature, we assumed that the higher affinity site is for lysine residue from the walker-A motif and moderate site is for serine residue from the signature motif. Considering this hypothesis, we initially expected a moderate affinity (1 μM) for the FtsE^K41A^ mutant. Unfortunately, we could not calculate the exact affinity of FtsE^K41A^ for ATP, as we could not achieve higher protein concentration due to its extensive sticky behavior. However, from the fluorescence binding experiment, we observed it to have weaker affinity compared with wildtype FtsE. The probable reason for this might be, binding of ATP at lysine residue enhances the second ATP binding affinity for serine residue. Thus the mutation of Lys^41^ to alanine could further reduce the affinity of FtsE for ATP at the second binding site Ser^139^. This could be the probable reason for not seeing the moderate binding of ATP in the FtsEK41A mutant.

Our study indicated that, like other NBDs, FtsE_Ec_ also contains two ATP-binding sites and follows the ATPase cycle. The ATPase cycle and its interaction with FtsE probably are regulating the PG hydrolysis at the mid cell by controlling the on/off switch of the amidases activation.

## Supplementary Material

Supplementary Figures S1-S4

## Data Availability

All supporting data are included within the main article and its supplementary files.

## Abbreviations

CD: circular dichroism
FtsE_Ec_: *Escherichia coli* FtsE
NBD: nucleotide-binding domain
PG: peptidoglycan
SEC: size-exclusion chromatography
TMD: transmembrane domain
Y2H: yeast two-hybrid
