# Supplementary material for: Biochemical characterization of an E. coli cell division factor FtsE shows ATPase cycles similar to the NBDs of ABC-transporters
Source: Biosci Rep. 2021 Jan 7;41(1):BSR20203034. doi: 10.1042/BSR20203034 (PMC7791547; doi:10.1042/BSR20203034)
Supplement: Supplementary Figures S1-S4 [file BSR-2020-3034_supp.pdf]

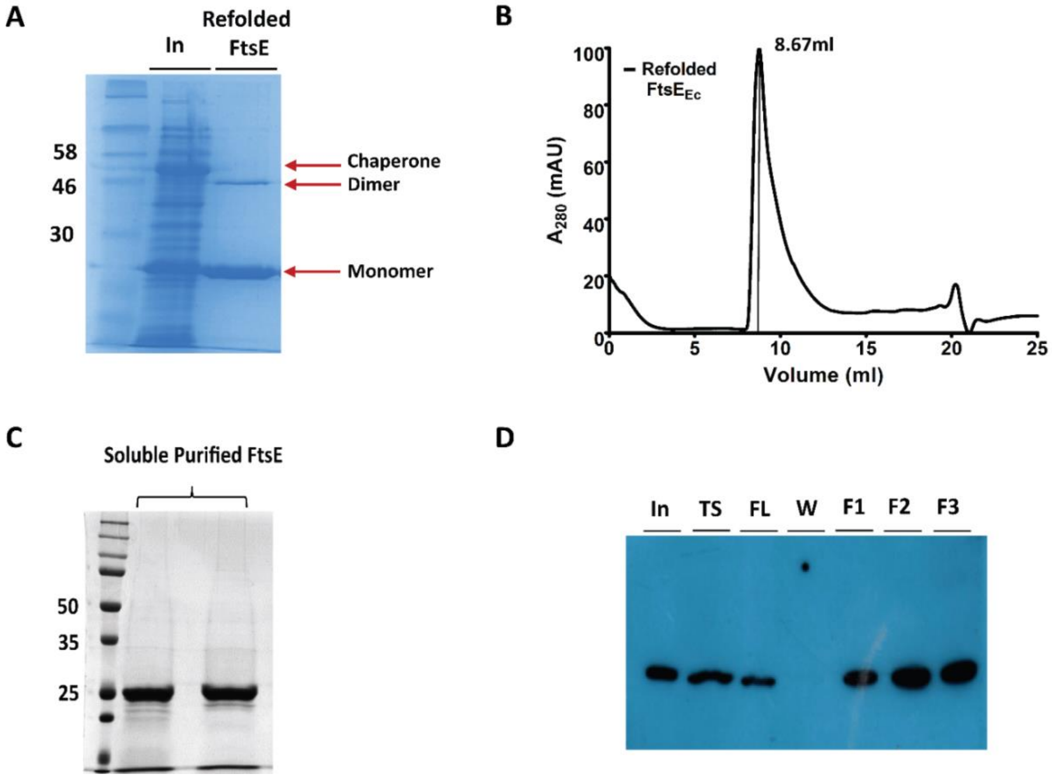

Fig. S1. Purified native and refolded FtsE.(A) Refolded and purified FtsE protein, the SDS PAGE image for refolded FtsE in 0.5M arginine, and purified from inclusion bodies. **(B)** Analytical size-exclusion chromatography profile for the refolded FtsE, eluting near to the void volume by using Superdex 200 10/300 GL column. **(C)** SDS PAGE image for soluble final purified FtsE. **(D)** Western blot with the anti-His antibody of FtsEduring steps of checking solubility and purification. In: Induced, TS:Total supernatant, FL:Flowthrough,W:Wash fraction, F1: Elution fraction 1, F2: Elution fraction 2, F3: Elution fraction 3.

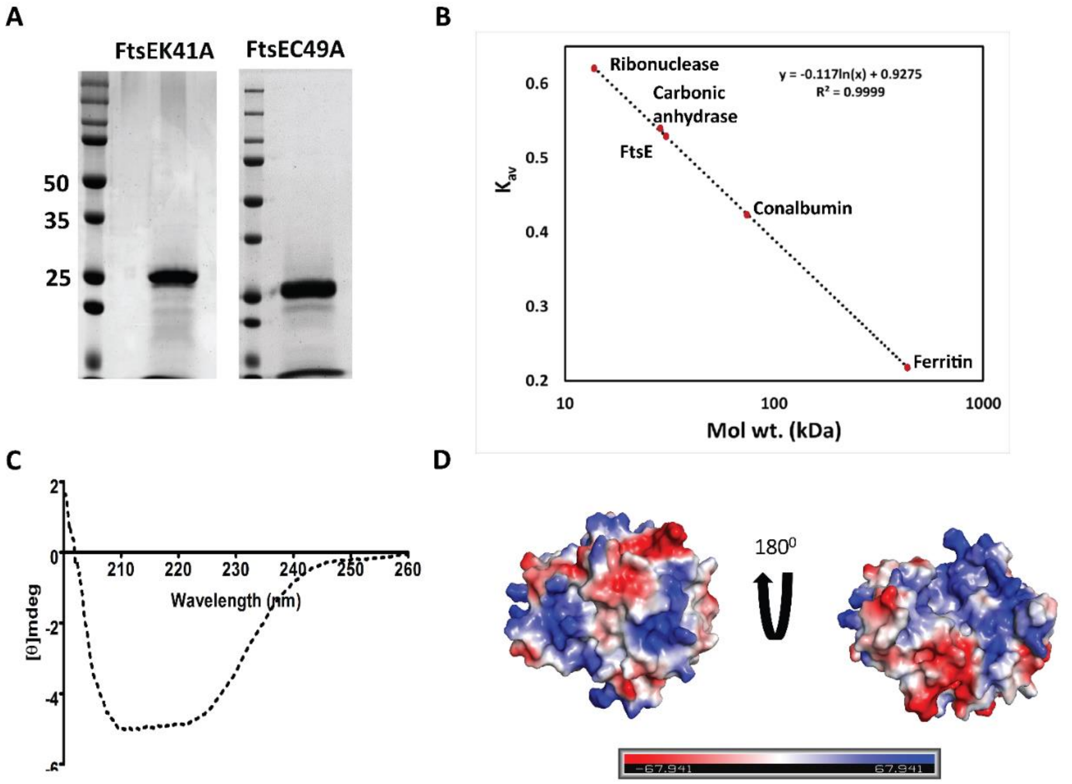

Fig. S2. Molecular weight calculation, CD spectra and surface charge distribution of FtsE. (A) The SDS-PAGE image of final purified mutant FtsE proteins from the soluble fraction. **(B)** Molecular weight calculation of the final purified soluble FtsE by following the equation described in materials and methods. **(C)** CD spectrum of FtsE obtained in soluble form and purified revealing proper secondary structure and hence folding with approximately 36.9% helix, 28.9% beta-sheet, and 5.3% turn. **(D)** Surface charge distribution of modeled FtsE.

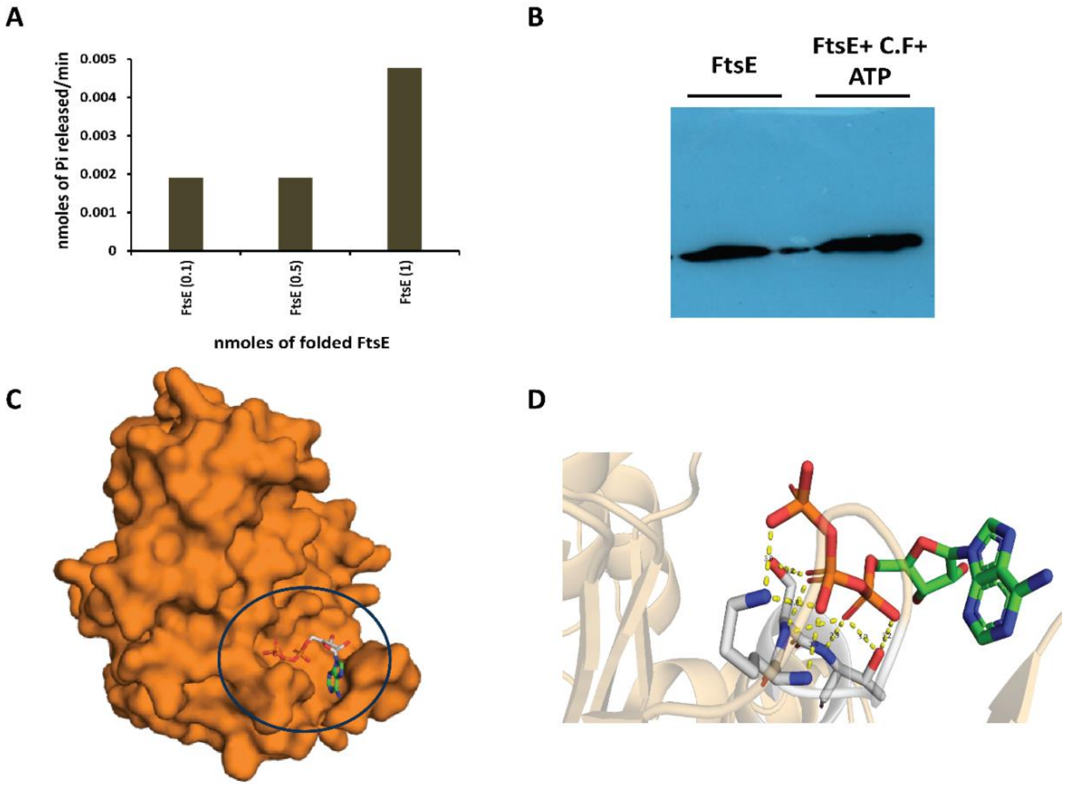

Fig. S3. ATPase activity of refolded FtsEand in silico interaction of ATP with FtsE. (A). Dendrogram showing the ATPase activity of refolded FtsE. **(B)**Western blot with the anti-His antibody showing the absence of dimerization of FtsE in the presence and absence of the bacterial cytoplasmic fraction. **(C)**Docking of FtsE modeled structure with ATP, showing its interaction with K41 residue.(D) Enlarged view of FtsEwith the docked ATP shows the interaction of lysine residue from the Walker-A motif and all the phosphate groups of ATP.

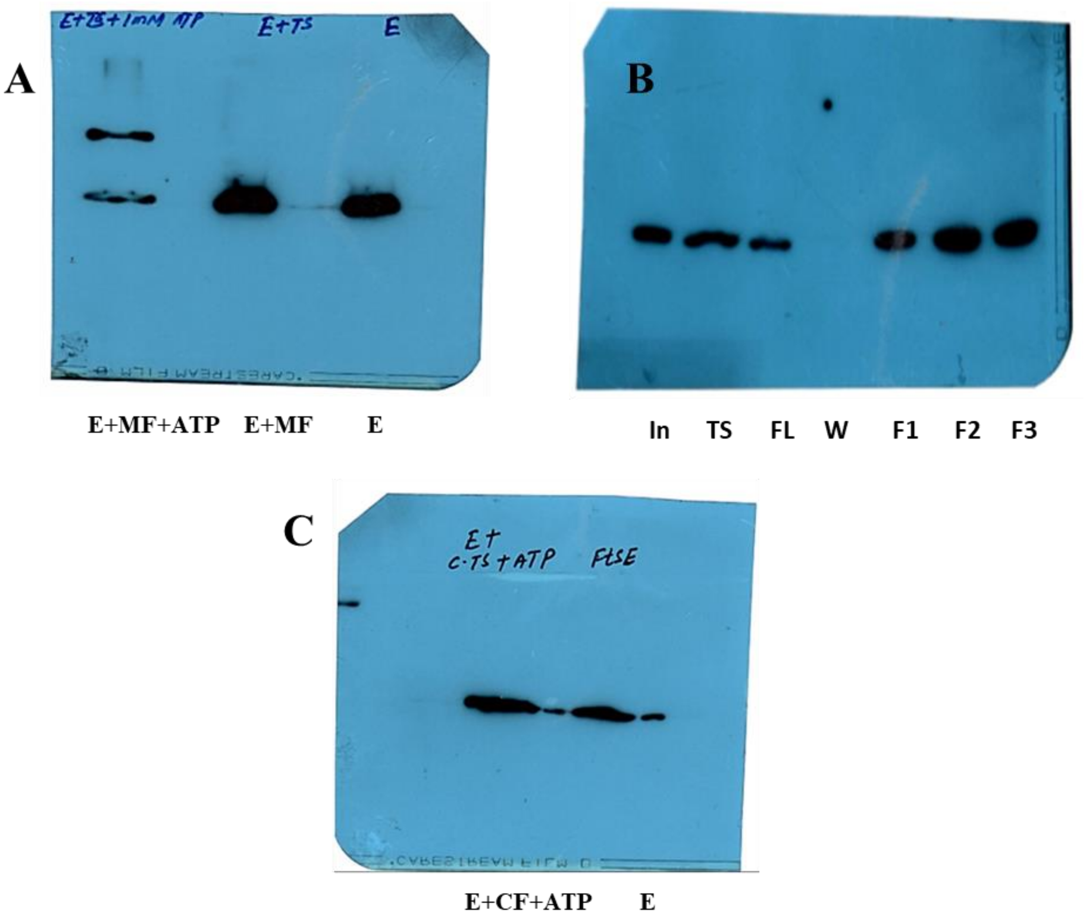

Figure S4. Uncropped western blots shown in the manuscript.***Panel A***shows the original westernblot, from which Fig. 5A was made. ***Panel B and panel C*** show the western blot from which S1D and S3B were made. All lanes shown in the western blot were used.
